# Physicochemical properties, texture, and probiotic survivability of oat‐based yogurt using aquafaba as a gelling agent

**DOI:** 10.1002/fsn3.1932

**Published:** 2020-10-06

**Authors:** Vassilios Raikos, Lina Juskaite, Frazer Vas, Helen E. Hayes

**Affiliations:** ^1^ Rowett Institute University of Aberdeen Foresterhill, Aberdeen UK

**Keywords:** aquafaba, consistency, oats, probiotic viability, texture, yogurt

## Abstract

Despite high consumer demands, the manufacture of nondairy yogurt from oat milk is currently hindered due to the lack of consistency and texture. An oat‐based yogurt was developed using oat milk and probiotics (*Streptococcus thermophilus* and *Lactobacillus bulgaricus*) with aquafaba (AF) and vegetable oil (VO) as added ingredients. Physicochemical analyses and viability of probiotics were investigated after yogurt formation and for 3 weeks under refrigerated storage. Results showed that adding AF decreased syneresis and increased water holding capacity during storage. Both AF and VO had a beneficial effect on hardness, the most important textural property of yogurt. Confocal laser scanning microscopy revealed that the added ingredients played a major role in the formation of the gel network structure of the yogurt. Both *Streptococcus thermophilus* and *Lactobacillus bulgaricus* remained at acceptable levels > 8.28 Log CFU/g and > 5.79 Log CFU/g after 3 weeks at 4°C regardless of the added ingredients.

## Graphical abstract

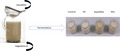

## 1 INTRODUCTION

Fermented dairy products contain essential nutrients and bioactive ingredients beneficial for human health, and therefore, their moderate consumption is recommended for the general population as part of a healthy eating regime (Wong et al., 2020). On the other hand, dairy products are responsible for several diet‐related adverse effects such as lactose intolerance and dairy protein allergy and thus may not be tolerated by an increasing segment of the population (Gupta et al., 2010). In addition, animal welfare and environmental issues are important societal drivers toward a “free from” diet which is predominantly associated with the consumption of plant‐based products.

Among nondairy milk substitutes, oat milk has recently attracted considerable attention. This is largely due to its agricultural performance as well as it nutritional profile. Oats (*Avena sativa* L.) is a competitive crop for arable production and a source of beneficial nutrients for human health including protein, starch, dietary fiber (β‐glucan), vitamins, and phytochemicals (Luana et al., 2014). The main beneficial effects of oats on human health are attributed to hypocholesterolemic and anticancerous properties exerted by several of its nutritional components. In addition, oats have also recently been considered suitable in the diet of celiac patients (Sontag‐Strohm et al., 2008).

Even though oat milk either as a whole or as a food ingredient in oat‐based foods such as breads, cookies, cereals, beverages, and biscuits has become increasingly popular, the manufacture of yogurt from oat milk remains challenging. This is largely due to the fact that fermented oat milk alone cannot form a gel network analogous to that found in traditional yogurt, which is widely appreciated by consumers for its sensory and textural properties (Walsh et al., 2010). The most commonly used methods to overcome this barrier in order to improve consistency and/or texture of yogurt include either the increase of total solids in the milk or the addition of functional ingredients acting as gelling agents or thickeners (Wang et al., 2012). Numerous ingredients have been used in the past to impart stability and improve texture in nondairy yogurts. These typically include hydrocolloids, and each one has its own benefits and limitations (McCann et al., 2011). Previous studies suggest that plant polysaccharides such as tragacanth gum can be suitable for certain food applications as thickeners or fat replacers but may have adverse or even detrimental effects on yogurt texture and overall quality (Ghaderi‐Ghahfarokhi et al., 2020).

Aquafaba is the wastewater derived from the cooking process of chickpeas, which is known to contain significant amounts of carbohydrates (predominantly starch), protein, and saponins. Aquafaba has documented ability to form emulsions and foams and shows potential as a gelling agent to improve texture in food formulations (Buhl et al., 2019). Furthermore, it can be used as a functional ingredient for the development of gluten‐free food products (Boucheham et al., 2019). The application of aquafaba in dairy‐free yogurt as a gelling agent shows potential yet remains largely unknown. The viability of probiotics during storage needs to be determined to ensure a minimum of 10^6^ CFU/g in order to achieve optimal potential therapeutic effects for the fermented product (Guo, 2007). Thus, the study of the structural, physical, and sensory properties of oat‐based yogurt enriched with aquafaba is an essential step toward understanding the products’ acceptability and shelf‐life. Therefore, the present work aims to investigate the physicochemical properties and probiotic survivability of oat milk yogurt using aquafaba as a gelling agent.

## 2 MATERIALS AND METHODS

### 2.1 Materials

Medium oatmeal (Gloagburn Farm) was used to make milk. Coconut oil blend was purchased from the local supermarket Sainsbury's and AF powder from VÖR (Vör Inc.). Yo‐Mix^®^ ABY yogurt culture (*Bifidobacterium lactis*, *Lactobacillus acidophilus*, *Lactobacillus delbrueckii subsp*. *bulgaricus*, *Lactobacillus delbrueckii* subsp. *lactis*, and *Streptococcus thermophilus*) and lactose food grade powder were obtained from Goat Nutrition Ltd. and Blackburn Distributions Ltd., respectively.

### 2.2 Preparation of oat milk yogurt

Oat milk (12% w/w oats) was prepared by adding 360 g oats into a container which was filled up to 3 L with water. The mixture was blended using a Robot Coupe Blixer 2 Mixer (Robot Coupe Ltd) for 1min. The mixture was then strained to extract the milk. Oat milk was used to prepare the following formulations (w/w): a. Control, b. 3% VO, c. 3% AF, and d. 1.5% VO‐1.5% AF. The milk with added ingredients was blended using a hand mixer to ensure homogenization. The samples were then placed in glass jars and were pasteurized using a Klarstein Biggie Digital fully automatic cooker at 90°C for 30 min by stirring the milk every 10 min to prevent lump formation. Samples were cooled in a cold tub until the temperature dropped below 50°C, and then, all jars were treated with 5% (w/w) lactose and 0.25% (w/w) Yo‐Mix^®^ ABY yogurt culture. Samples were fermented at 43°C for 6 hr in a 7‐Cup Electric Yogurt Maker (Lakeland, Aberdeen, UK) and stored in the fridge at 4°C overnight prior to analyses. A portable food and dairy pH meter (Hanna Instruments Ltd.) was used to measure the changes in pH of the samples before, during, and after the fermentation on an hourly basis to ensure a drop in pH. Three different batches were prepared at different days for all treatments to ensure consistency of the preparation method.

### 2.3 Proximate analysis of AF

Nonstarch polysaccharides (fiber) were determined by the Englyst procedure (Englyst et al., 1994), and fat was determined by the Bligh & Dyer method (1959) and nitrogen content by the Dumas combustion method (1831).

### 2.4 Water holding capacity (WHC)

WHC was determined by the method reported by Remeuf et al. (2003). 10 g of yogurt were centrifuged at 4,000 rpm for 30min at 4˚C. The expelled water was removed and weighed. The measurements were repeated on a weekly basis for 3 weeks. The percentage of WHC was calculated according to the equation:

(1) $$\text{WHC}=\left[\left(\text{Sample weight}‐\text{Expelled water}\right)/\text{Sample weight}\right]\times 100$$

### 2.5 Syneresis

50 g of yogurt samples were weighed onto a 2‐V folded filter paper (Whatman) and placed on the top of a funnel. Syneresis was determined by gravity by measuring the weight (g) of liquid collected in a volumetric flask of known weight. The drainage time and temperature were 120 min and 4˚C, respectively. The percentage of syneresis was calculated according to the equation:

(2) $$\text{Syneresis}\;\left(\%\right)=\left(A/B\right)\times 100$$

where *A* = weight of liquid collected and *B* = initial weight of sample (50 g).

### 2.6 Titratable acidity

Titratable acidity was measured by diluting 9 g of yogurt samples with an equal amount of Milli‐Q water. 1 ml of phenolphthalein was added and then the sample was titrated with 0.1 M NaOH, noting the volume used to turn the sample pink in color. Titratable acidity was then calculated and expressed as percent lactic acid as follows:

(3) Lactic acid%=V×0.009/W×100

where *V* is the volume of 0.1 M NaOH (ml) and W is the weight of yogurt (g).

### 2.7 Texture analysis

Texture measurements were performed using a CT3 Texture Analyser (Brookfield Engineering Laboratories Inc.) and a cylindrical probe (TA4/1000, 38.1 mm diameter). Data were recorded using Texture Proc CT V1.3 Build 15 software (Brookfield Engineering Laboratories Inc.). Yogurt samples (150 g) were poured in a cone baker and were analyzed using the following 2 cycle compression test settings: target distance = 30.0 mm; trigger load = 10 g; test speed = 1.00 mm/s; and return speed = 1.00 mm/s. The following parameters were recorded: hardness, adhesiveness, cohesiveness, chewiness, gumminess, and springiness.

### 2.8 Confocal laser scanning microscopy

Images were captured on a Carl Zeiss LSM 710 inverted confocal microscope using an ×40 oil objective lens. Images were captured using 488 nm laser set at 2% and frame size 1024 × 1024. Rhodamine B (Sigma‐Aldrich Ltd) was dissolved in distilled water at 1 g/L. This dye was used to stain proteins and starch (λexmax 488 nm, λemmax 657 nm). Nile red (Sigma‐Aldrich) was dissolved in propylene glycol (Sigma‐Aldrich) at 1.2 g/L. This dye was used to stain fat (λexmax 543 nm, λemmax 561 nm). 1ml of sample was stained with 10 μl of the rhodamine B solution and 10 μl of the Nile red solution.

### 2.9 Lactic acid bacteria viability

Samples were withdrawn weekly from all treated samples to determine the survivability of probiotics. Colony enumeration was carried out after serial dilutions of the yogurt at days 1, 8, 15, and 22 at 4°C using a method described previously (He Ni et al., 2018). *Lactobacillus bulgaricus* was inoculated in Lactobacillus selective agar (83920 Rogosa Agar, Sigma‐Aldrich) and incubated at 37°C for 4 days, and *Streptococcus thermophillus* was cultivated in M‐17 agar (Sigma‐Aldrich, Dorset, UK) and incubated at 37°C for 2 days.

### 2.10 Statistical analysis

All experiments were conducted on at least one replicate from each batch (*n* ≥ 3). Results are expressed as means ± standard deviation (*SD*) of at least three replicates. Data were subjected to statistical analysis by SPSS Statistics 25 software. The normality of data distribution was tested by Shapiro–Wilk method. Statistical significance values of groups’ means were made by analysis of variance (ANOVA) for day 1 measurements and repeated measure analysis of variance (rm ANOVA) for repeated measurements (day 1‐day 22). The Bonferroni post hoc test was used to detect statistically significant results. The statistical analysis performed was considered significant when *p* < .05.

## 3 RESULTS AND DISCUSSION

Despite the fact that oats contain significant amounts of protein (predominantly globulins), previous attempts to develop a yogurt‐style product without fortification with gelling agents proved challenging and were characterized by the formation of a weak gel with increased syneresis (Deswal et al., 2014; Walsh et al., 2010). This is because the microstructure of the gel network formed in typical dairy yogurt formulations is owed primarily to dairy proteins. Casein particle aggregation occurs upon acidification and the interaction of the later with denatured whey protein results in increased gel firmness and viscosity (van Vliet et al., 2004). Oats typically contain 60% starch, 11%–15% total protein, 5%–9% lipids, 2.3%–8.5% dietary fiber, and 0.54% calcium (Rasane et al., 2015). The industrial process for oat milk manufacture involves starch hydrolysis with α‐amylase to prevent starch gelatinization during pasteurization of the product (Deswal et al., 2014). In the present study, the enzymatic degradation of starch was eliminated from the process of milk making from oats. The rationale was to preserve the structure of starch and induce gelatinization during thermal processing and fermentation. Starch gelatinization increases the viscosity of the system through the “filler effect” attributed to swollen starch particles which absorb water and interact with other particles in their vicinity thus leading to an increase in the starch phase volume (Lobato‐Calleros et al., 2014; Pang et al., 2019).

**FIGURE 1:**
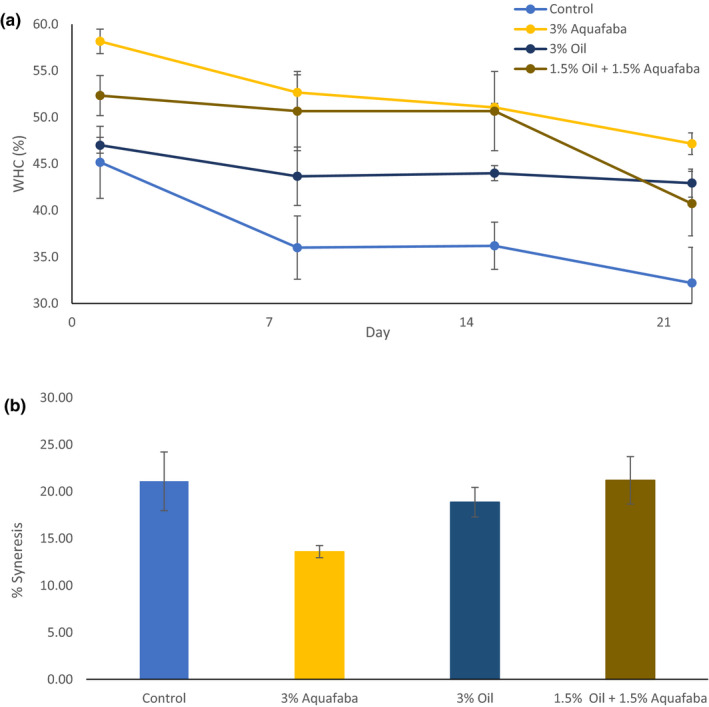
Formulation effects on % WHC (a) and syneresis (b) of oat‐based yogurt

**FIGURE 2:**
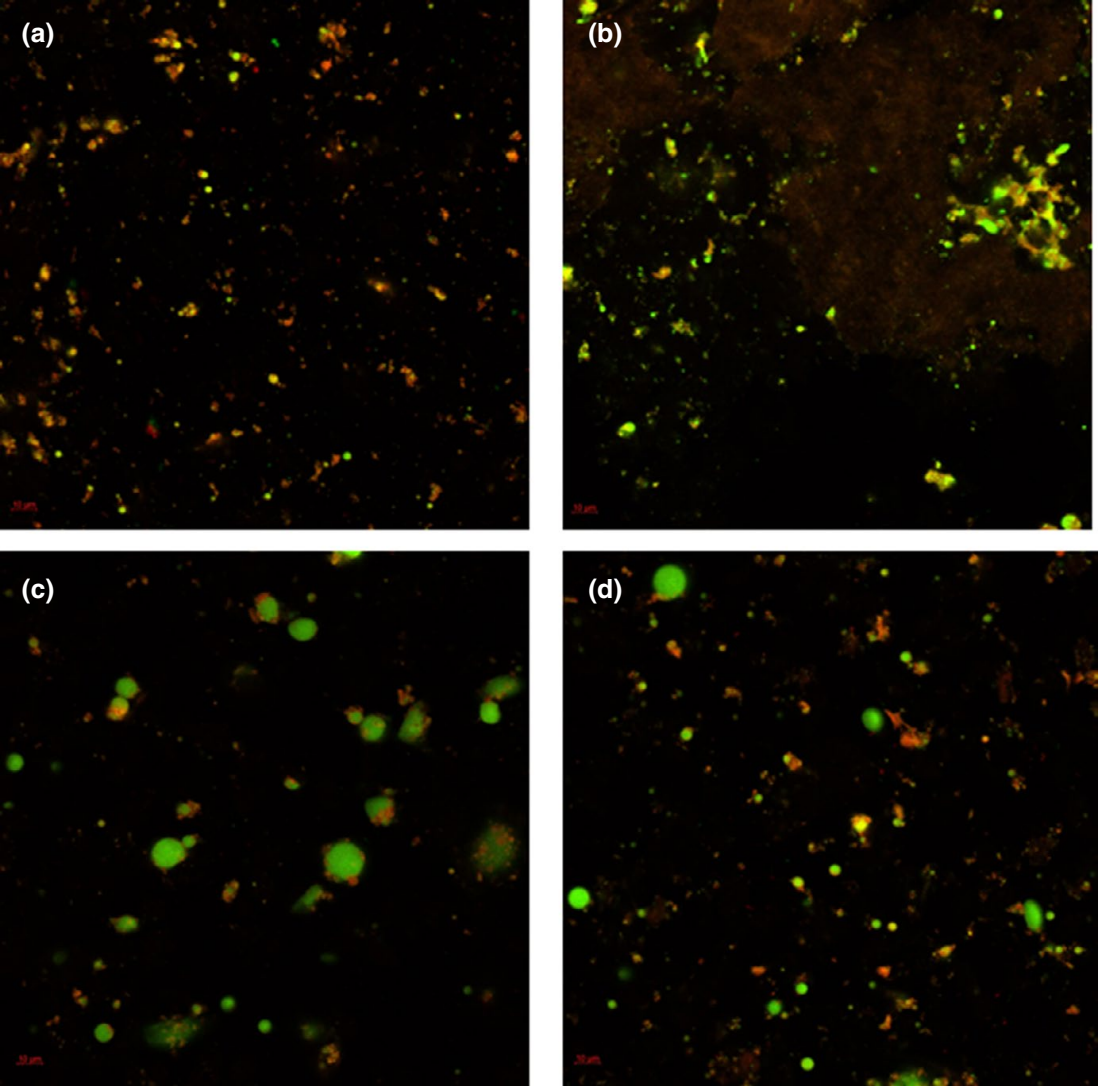
Confocal laser scanning microscopy of yogurt samples stained with Rhodamine B and Nile Red: (a) Control, (b) 1.5% AF, (c) 1.5% Oil, (d) 1.5% AF + 1.5% Oil. Scale bar is 10 μm

Oat milk (12%) was used as the primary ingredient for developing a yogurt formulation by following the standard steps involved in yogurt manufacture and AF was used as a gelling agent to improve consistency and texture. Whey separation is an important defect in yogurt and can be defined as the appearance of whey on the gel surface of set‐type yogurts. Syneresis is the shrinkage of the gel, which then leads to whey separation (Lucey, 2004). The water holding capacity (WHC) of yogurts during 3 weeks of storage at 4°C and syneresis data from the freshly prepared samples are presented in Figure 1. Results showed that the addition of AF (3%) decreased the level of serum loss from 21ml for the control to approximately 14ml. Formulations which contained vegetable oil alone or in combination with AF were less favorable in reducing serum separation. Similarly, water holding capacity of the gel formed at day 1 was highest for the formulations containing AF and followed the order: 3% AF (58%) > 1.5% vegetable oil + 1.5% AF (52%) > 3% vegetable oil (47%) > control (45%). There was a significant (*p* = .002) effect for storage time on WHC which indicates syneresis and accumulation of serum on the gel surface after 3 weeks at 4°C. However, the reduction in WHC with time is independent on the yogurt additives as all samples exhibit similar reduction in WHC over time. The beneficial effect of AF in reducing syneresis and increasing WHC is attributed to the significant increment in the total solids contributed by the protein and fiber content of the added by‐product (Table 1). A contributing factor to the desired consistency of yogurt is the total solids content (~12–15 g/100 g), total protein content, and type of milk (Domagala, 2009). In the present work, the contribution of the added AF to the total solids is considered significant due to the fact that the fortificant was used in a dried (condensed) form. Data from previously published work suggest that the increment of total solids and the addition of fibers can enhance water entrapment within the gel network and reduce syneresis (McCann et al., 2011; Rinaldoni et al., 2012). Furthermore, the formation of an extended gel network due to the ionic interaction between proteins from AF and carbohydrates and the subsequent formation of aggregates via hydrogen bonding, capable of immobilizing water can also account for the observed beneficial effect of AF on WHC and syneresis (Walsh et al., 2010). This is further supported by the examination of the microstructure of the yogurts (Figure 2). Solubilized starch granules and proteins (stained with Rhodamine B and shown as red) are the main structuring units in the form of small aggregates in yogurt prepared without any fortificants (2A). Proteins and starch granules form a dense gel network, which seems to be more extended in the presence of AF (2B). The addition of fat (stained with Nile Red and shown as green) in the form of well‐defined spherical particles seems to be contributing less to the formation of the gel network (2C).

**TABLE 1:** Proximate composition of dried aquafaba broth

% (weight)	Protein	Total fat	Fiber
Aquafaba	20.2 ± 0.04	0.13 ± 0.03	4.04 ± 0.09

Texture profile measurements of the oat‐based yogurts are presented in Table 2. Hardness is the most important textural parameter for yogurt evaluation and is considered a valid indicator of the products’ firmness (Mudgil et al., 2017). The peak force required to fracture the gel was higher for yogurt formulations containing AF or oil (3%). Fat plays an important role in controlling the firmness and perceived creaminess of yogurt due to the formation of a large number of small, spherical particles upon interaction with the protein matrix (McCann et al., 2011). Yogurt samples without additives exhibit the lowest hardness, which suggests that fortification resulted in the formation of a gel with enhanced density and strength. On the other hand, adhesiveness, which is a measure of stickiness of yogurt and is inversely related to consumer acceptability, was higher in yogurt samples with AF. The inclusion of vegetable oil in oat‐based formulation seems to have a beneficial impact on the textural properties of yogurts.

**TABLE 2:** Textural properties of oat‐based yogurt with and without additives

	Control	3% Aquafaba	3% Oil	1.5% Oil + 1.5% Aquafaba
Hardness	25.50 ± 1.15	32.00 ± 3.09	32.17 ± 2.43	27.33 ± 0.27
Adhesiveness	1.80 ± 0.31	2.33 ± 0.24	1.87 ± 0.23	1.73 ± 0.33
Cohesiveness	0.67 ± 0.05	0.60 ± 0.03	0.69 ± 0.03	0.65 ± 0.05
Springiness	27.47 ± 0.28	27.74 ± 0.89	27.63 ± 0.20	27.04 ± 0.42
Gumminess	16.83 ± 1.28	20.33 ± 2.23	21.83 ± 1.32	19.33 ± 1.96
Chewiness	4.57 ± 0.37	7.44 ± 1.07	5.90 ± 0.33	6.78 ± 0.93

**FIGURE 3:**
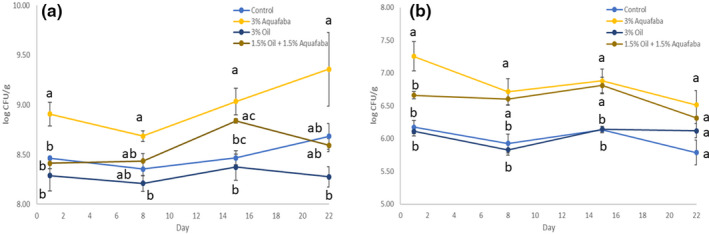
Viable counts of *S. thermophillus* (a) and *L. bulgaricus* (b) in oat‐based yogurts during 3 weeks of storage at 4°C. Each point is the mean of 2 replicates from each batch (*n* = 6) ± *SD*

The viability of lactic acid bacteria was at acceptable levels after fermentation and remained relatively stable over the 3‐week storage period regardless of fortification (Figure 3). A beneficial effect in probiotic growth was observed in samples containing AF which was significant (*p* < .05) compared with control at different time points for both *Lactobacillus bulgaricus* and *Streptococcus thermophillus*. The counts of viable *Lactobacillus bulgaricus* decreased significantly (*p* < .05) with storage time, and the rate of this loss was dependent on the yogurt formulation. Previous research has indicated that fortification of yogurt with chickpea flour stimulated the growth of probiotic bacteria and maintained higher counts than the control over the 21‐day refrigerated storage (Chen et al., 2012). The observed prebiotic effect of aquafaba is largely attributed to the mineral (zinc and iron) and oligosaccharide composition of chickpea (Zare et al., 2012). The pH of yogurts fortified with AF was significantly higher compared with that of the control after 6h of fermentation (Table 3). This effect has been previously documented and is attributed to fiber's chemical composition (Hashim et al., 2009). Furthermore, acidity levels were also significantly higher for samples fortified with AF. Previous studies have shown that oat‐fiber fortified yogurts contained significantly higher levels of acetic and propionic acids (Fernández‐García et al., 1998). The fortification of yogurt with fiber with prebiotic effects has been documented to increase the metabolic rate of yogurt starter culture (Yetka & Ansari, 2019). Thus, this effect may be attributed to increased metabolic activity of lactic acid bacteria in samples fortified with AF, which confirms the viability data obtained.

**TABLE 3:** Titratable acidity and pH of oat‐based yogurts

	Control	3% Aquafaba	3% Oil	1.5% Oil + 1.5% Aquafaba
% Titratable acidity	0.19 ± 0.01^a^	0.50 ± 0.03^b^	0.19 ± 0.02^a^	0.37 ± 0.02^c^
pH	3.9 ± 0.01^a^	4.2 ± 0.01^b^	3.9 ± 0.01^a^	4.1 ± 0.01^c^

Different low case letters indicate significant differences between mean values for each treatment (*p* ≤ .05).

## 4 CONCLUSIONS

The addition of aquafaba as a gelling agent for the development of oat‐based yogurt was investigated. Aquafaba had a beneficial effect in increasing water holding capacity and decreasing syneresis. Yogurts formulated with vegetable oil acquired more desirable textural properties. Probiotic viability was significantly increased by the addition of aquafaba and remained at therapeutic levels (>7 log cfu/g) during the storage period for all treatments. Aquafaba can be used as a gelling agent to improve consistency of nondairy fermented products. The main limitation of the study was the batch variability between samples which reduced the statistical power of the data. Further work needs to be conducted to standardize the process and optimize yogurt formulations.

## CONFLICTS OF INTEREST

The authors declare that there are no conflicts of interest.
